# Validation of the Italian version of the Short Mood and Feelings Questionnaire (SMFQ) in a sample of adolescents

**DOI:** 10.1186/s13052-026-02240-7

**Published:** 2026-03-21

**Authors:** Luisa Almerico, Anna Pezzella, Carmela Bravaccio, Raffaella Iuliano, Gennaro Catone, Simone Pisano, Vincenzo Paolo Senese

**Affiliations:** 1 Department of Psychology, University of Campania “Luigi Vanvitelli”, Caserta, Italy; 2 Department of Translational Medical Sciences, University “Federico II”, Naples, Italy; 3 UOSD Neonatology, “Ospedale del Mare”, ASL NA 1, Naples, Italy; 4 Department of Educational, Psychological and Communication Sciences, “Suor Orsola Benincasa” University, Naples, Italy

**Keywords:** Adolescence, Depression, SMFQ, Validation, Measurement invariance

## Abstract

**Background:**

Depression is a prevalent and often persistent mental disorder, with onset frequently occurring during adolescence and associated with serious long-term consequences. Consequently, the availability of valid, age-appropriate, and easily administered screening instruments is essential. The Short Mood and Feelings Questionnaire (SMFQ) is a widely used tool, available in multiple languages, for the screening of depressive symptoms in young people. Although an Italian version of the SMFQ has already been used in previous studies, it has not yet undergone formal validation. The present study aimed to evaluate the psychometric properties of the Italian SMFQ, assess measurement invariance (MI) across sex and age, and provide preliminary normative data.

**Methods:**

A sample of 580 adolescents (317 males, 263 females), aged 14–20 years, completed the Italian SMFQ along with additional instruments to assess validity: the Personality Assessment Questionnaire and the Strengths and Difficulties Questionnaire for convergent validity, and the Weinstein’s Noise Sensitivity Scale for divergent validity. Analyses focused on dimensionality, measurement invariance, reliability, and validity.

**Results:**

As regards dimensionality, both one- and two-factor models showed adequate fit, with the two-factor model showing better fit. MI analyses supported full invariance across age for the 13-item version and partial invariance across sex. The 12-item version (excluding item 6) achieved full invariance across both sex and age. The scale showed good internal consistency and good convergent and divergent validity. Finally, given the significant effects of sex and age, normative data were computed as a function of both factors.

**Conclusions:**

The Italian SMFQ demonstrated excellent psychometric properties for assessing depressive symptoms in adolescents. However, its use for clinical diagnostic purposes requires further validation through clinical studies.

**Supplementary Information:**

The online version contains supplementary material available at 10.1186/s13052-026-02240-7.

## Introduction

Depression is a prevalent mental disorder worldwide, characterized by persistent sadness, loss of interest, changes in weight and sleep, increased fatigue, feelings of worthlessness or guilt, and suicidal ideation [1–2]. It frequently emerges during adolescence [3], with at least half of lifetime cases reporting a first episode around the age of 14 [4]. This developmental period involves profound biological, cognitive, and social changes that heighten sensitivity to social stimuli [5–8] and increase vulnerability to internalizing disorders, particularly anxiety and depression [9–10]. Despite its prevalence, adolescent depression often remains undetected or untreated [11], as symptoms may be misinterpreted as normative developmental stressors or transient phases [12], with possible negative outcomes [11–13].

The recent pandemic has further increased its prevalence among adolescents worldwide [14–15], underscoring the need for screening tools that are easy to administer, valid and reliable. Self-report measures represent a valuable and efficient approach for large-scale screening, allowing adolescents to articulate their internal experiences in a structured format with minimal resource requirements [16].

The MFQ [17] is a 33-item screening scale used in clinical and research settings to identify depressive symptoms in children and adolescents aged 8–18 years. It has been validated against DSM-IV and ICD-10 diagnoses [18–19] and is recommended by the National Institute for Health and Clinical Excellence [20] for screening purposes [21–22]. A shorter yet validated version, the SMFQ [23], consists of 13 items assessing depressive symptoms over the past two weeks in individuals aged 8–18 years. Developed from core MFQ items [23], the SMFQ is widely used due to its brevity and suitability for epidemiological research. It effectively differentiates clinically depressed from non-depressed individuals [24–26] and has been validated in both clinical (e.g. [24, 27, 28]), and nonclinical samples (e.g. [26, 29]),. Although translated into multiple languages [30–35], evidence regarding its psychometric properties in Italy remains limited.

The factorial structure of the SMFQ has been extensively investigated in samples aged 6–25 years. Several studies support a unidimensional solution [24, 29, 31, 33], whereas Lewis et al. [36], analysing Australian, American, and Indian samples, proposed two correlated factors: an affective/somatic dimension (including item 1, 2, 3, 4, 5, and 7; e.g., “Didn’t enjoy anything”, “So tired I did nothing”), and a cognitive dimension (including item 5, 6, 8, 9, 10, 11, 12, and 13; e.g., “I was a bad person”, “Nobody really loved me”). This distinction is consistent with neurobiological evidence linking affective symptoms to dysfunctions in emotional regulation areas, while cognitive symptoms to executive functioning and cognitive control dysfunctions [37–39]. However, the strong correlation between the two factors (*r* =.45–0.49) suggests an overarching general depression construct. Construct and criterion validity have also been supported [26, 29, 40], and reliability has been consistently reported across different age cohorts [23, 25, 29, 41].

Although the SMFQ’s psychometric properties have been widely studied, less attention has been given to its measurement invariance (MI), which examines whether the scale assesses depression consistently across groups or over time [42]. Establishing MI ensures that score differences reflect true variations in depressive symptoms rather than measurement bias [42]. A recent longitudinal study by Schlechter et al. [43] examined the SMFQ’s invariance across sex and age in a large sample of participants aged 11 to 26 years. The results supported age invariance and partial sex invariance, with differences emerging on the item “I cried a lot” (item 6), where females scored higher than males. These findings highlight the need to consider item-level differences, which may be influenced by cultural factors [44–46], while supporting the overall validity of the SMFQ across populations [47–48]. Although the SMFQ has been widely used internationally and applied in Italian through back-translation procedures [14], its psychometric properties in the Italian context have not been systematically examined.

Based on these considerations, the present study aimed to verify the preliminary psychometric properties of the Italian version of the SMFQ [14]. The research objectives were threefold. The first was to define the factorial structure of the scale. Considering discordant data in the literature, both the one- and two-factor structure were tested [24, 29, 36]. The second objective was to assess measurement invariance across sex and age. The third was to evaluate reliability and construct validity. Convergent validity was examined using the Personality Assessment Questionnaire (PAQ; [49–51]) and the Strength and Difficulties Questionnaire (SDQ; [52]), whereas discriminant validity was assessed with the Weinstein’s Noise Sensitivity Scale (WNSS; [53]). The SMFQ was expected to demonstrate solid psychometric properties and good construct validity, invariance across age, and partial invariance across gender for items related to unhappiness or crying [31, 43]. Additionally, the influence of age and sex on SMFQ scores was explored through hierarchical multiple regression, and normative reference values by age and sex were established. These reference values, expressed as expected means and percentile thresholds, are intended to support interpretation in research and applied settings. In the absence of clinical diagnostic criteria, the study focused on psychometric evaluation rather than diagnostic accuracy.

## Methods

### Participants

In this study, we combined the data collected in the study by Pisano and colleagues [14] from April to May 2020, with another independent sample of students collected between May and June 2020. Both samples were collected during the same phase of the COVID-19 pandemic in Italy. For each of the two data collection, a convenience sampling method was employed, while striving to ensure that participants within age-based groups were as balanced as possible in terms of gender, school and cities. Participants were recruited from four schools located in both the metropolitan and peripheral areas of Naples, in the south of Italy. All schools were professional institutes, which in the Italian educational system are typically attended by students from low- to middle-income backgrounds. While no specific data on ethnic composition were collected, the schools serve predominantly Italian adolescents. The resulting sample consisted of 580 Italian students aged between 14 and 20 (*M* = 16.4 years; *SD* = 1.5). These included 317 males (54.7%, with a *M* age of 16.1 years and a *SD* = 1.5) and 263 females (45.3%, with a *M* age of 16.7 years and a *SD* = 1.5). Before starting data collection, both parents and adolescents were required to provide consent for participation via an online informed consent form.

### Procedures

Participants were selected through convenience sampling from four schools in the city of Naples, Italy. Initially, schools provided detailed information about the research to both adolescents and their parents, allowing them to address any queries with teachers or researchers. Subsequently, schools provided their students with a link to the online platform to access the survey. Data were collected through the Qualtrics online survey platform during a period of remote schooling, so students completed the survey from home but were supervised in real time by a research assistant during a scheduled virtual classroom session. Participants completed a protocol comprising three sections: the socio-demographic questionnaire (to gather information such as age, gender, level of education), the SMFQ, and the criterion measures. The first two sections of the protocol were administered to all participants. To assess the convergent validity of the SMFQ, a randomly selected subsample of participants (*n* = 313) was administered the “emotional symptoms” subscale of the SDQ [52]; whereas another randomly selected subsample of participants (*n* = 267) completed both the PAQ [49–51], to test convergent validity, and the WNSS scales [53], to assess divergent validity. All the measures were completed in a single session and the SMFQ was always presented prior to the criterion measures to minimize potential response bias.

### Measures

#### Short Mood and Feelings Questionnaire

The SMFQ was administered to evaluate affective and cognitive symptoms associated with depression [23]. This self-report scale comprises 13 items, adapted from the 33-item version of the MFQ [17]. Each item is assessed on a three-point scale, with responses of “True” (= 2), “Sometimes True” (= 1), and “False” (= 0), yielding a total score ranging from 0 to 26. Higher scores indicate greater severity of depressive symptoms. In this study, we used the Italian version of the SMFQ adapted into Italian by Pisano and colleagues [14]. For the full questionnaire, including instructions in Italian, see Additional File – Appendix 1.

#### Personality assessment questionnaire

The short form of the Italian Adult PAQ ([49–51, 54]) was administered to assess the general psychological adjustment [55–57]. The self-report scale consists of 42 items divided into seven domains (6 items for each domain): hostility/aggression, dependence or defensive independence, negative self-esteem, negative self-adequacy, emotional unresponsiveness, emotional instability and negative worldview. For each item, participants are asked to reflect on their feelings about themselves. Individuals can respond to items on a 4-point-scale ranging from “almost always true of me” (= 4), to “almost never true of me” (= 1), with higher scores indicating less positive psychological adjustment. In line with the literature [49–51], in this study we have considered only the total score of psychological maladjustment, that can be computed by summing the total score of all the sub-scales except for the “dependence or defensive independence” dimension (Cronbach α = 0.797).

#### Weinstein’s noise sensitivity scale

The Italian Version of the WNSS [53] was administered to assess individual differences in sensitivity to noise. The scale is a unidimensional, self-report measure of sensitivity to noise composed of 20 items addressing affective reactions and attitudes to both general noise and daily environmental sounds. For each statement, participants must indicate their agreement by means of a 6-point Likert scale, ranged from “disagree strongly” (= 1) to “agree strongly” (= 6). Agreement with the item indicates greater noise sensitivity of the respondent (Cronbach α = 0.756).

#### Strength and difficulties questionnaire

The SDQ [52] is a self-report measure used to assess general psychopathology. It consists of 25 items related to five domains: emotional symptoms, conduct problems, hyperactivity-attention problems, peer problems and prosocial behaviour. The items are rated on a 3-point scale from “not true” (= 0) to “certainly true” (= 2). For the present study, we used the “internalising” subscale score as a measure of internalising psychopathology referring to the last six months. This score was computed by summing the Emotional Symptoms and Peer Problems subscales, both ranging from 0 to 20. Higher scores indicated greater internalising psychopathological symptoms (Cronbach α = 0.733).

### Statistical analyses

Firstly, the main descriptive statistics were computed. Subsequently, statistical analyses were conducted to explore the psychometric properties of the SMFQ. Specifically, confirmatory factor analysis (*CFA*) was employed to investigate both the unidimensional and two-dimensional structure of the SMFQ, and then measurement invariance across sex and age was conducted. Finally, correlations between the SMFQ, PAQ, SDQ-Internalising symptoms subscale and WNSS were computed. *CFA* and measurement invariance analysis were performed using Mplus software (version 8.1; [58]) that is particularly suited for categorical data especially in the psychometric field. All other statistical analyses were carried out utilizing R software (version 4.3.1; [59]) We included only the respondents who completed all items of the protocol. For all statistical tests, significance level was set at α = 0.05.

#### Confirmatory factor analysis

Two confirmatory factor analyses (*CFA*) were carried out to evaluate the factorial structure of the SMFQ. Specifically, we compared both the 1-factor model and the 2-factor model proposed by Lewis et al. [36]. In the latter model, two correlated factors were considered: the first dimension (F1) related to the “affective-somatic” dimension, included items 1, 2, 3, 4, 5, and 7, while the second dimension (F2) related to the “cognitive” dimension, included items 8, 9, 10, 11, 12, and 13. Considering findings from previous studies [43, 44], and in line with the results from the modification indices analysis, we decided to allow the covariance between item 1 and item 6 to be freely estimated. Due to the ordinal nature of the SMFQ items, the Diagonally Weighted Least Squares (WLSMV) was used for parameter estimation. To assess model fit, we used the robust Chi-square test (SBχ^2^) and other fit indices that are less dependent on sample size [60]: the root mean square error of approximation index (*RMSEA*) and the Standardized Root Mean Square Residual (*SRMR*) as absolute fit indices; the comparative fit index (*CFI*); the nonnormative fit index (*NNFI*) as incremental fit index. For SBχ^2^ test, values associated with *p* >.05 were considered well-fitting models. For the other indices, we referred to Hu and Bentler’s [61] guidelines: *RMSEA* values less than 0.08 were deemed acceptable, with values below 0.06 indicating good fit; *RMSR* values below 0.08 were considered adequate; *CFI* and *NNFI* values above 0.90 were indicative of adequate model fit. To compare the relative fits of the nested models, differences in chi-square statistics (ΔSBχ^2^) and in *CFI* values (Δ*CFI*) were computed. In particular, the DIFFTEST function with the *WLSMV* estimator was used, which in this case defaults to the robust chi-square difference test described by Satorra and Bentler [62], taking into account the potential non-normality of data distributions when comparing the fit of nested models. This test assessed whether adding parameters to a more parsimonious model (nested model) significantly improved its fit compared to a more complex model (reference model). If the difference in robust chi-square statistics (ΔSBχ2) was significant with *p* <.05, or if it had a Δ*CFI* > 0.01 [63], it was interpreted as a reduction in fit, and the more constrained model was rejected. Otherwise, the more constrained model was accepted and considered as a best fitting model.

#### Measurement invariance

MI was tested across sex and age following the guidelines suggested by Putnick and Bornstein [42]. Specifically, we tested configural, metric, scalar, and residual invariance. To test MI across sex, two groups were compared, males (*n* = 317) and females (*n* = 263), while to test MI across age, two groups were compared, G1 (14–16 years, *n* = 293) and G2 (17–20 years, *n* = 287). Due to the categorical nature of the observed variables, also in this case the *WLSMV* was used. The same goodness-of-fit indices and test as the *CFA* were used to verify invariance of models. If the difference between the reference model and the more constrained model was significant it was interpreted as a reduction in fit, and the constrained model was rejected; otherwise, it was accepted and considered the new reference model. If the constrained model was rejected, a less restrictive model of partial invariance was evaluated in which, in accordance with modification indices and analysis of parameter estimates, equality constraints on one or more items were relaxed. If the model of partial invariance was accepted using these criteria, it was considered as the new reference model; otherwise, fitting more constrained models was suspended, and the previous reference model was interpreted as the final model expressing the highest hierarchical level of MI.

#### Reliability

Both Cronbach’s alpha (α) and omega (ωt) for ordinal measures [64] were used to test the reliability of the SMFQ scores.

#### Construct validity

Pearson’s correlation coefficients were computed to evaluate the nomological validity of the SMFQ scale. In particular, the correlation between the SMFQ scores and both the PAQ total score and the “internalising” subscale of the SDQ were computed to evaluate the convergent validity of the SMFQ; while the correlation between the SMFQ total score and the WNSS score was computed to evaluate the discriminant validity.

#### Effect of sex and age on SMFQ scores

To examine the impact of invariance factors (sex and age) on self-reported depressive symptoms, a hierarchical multiple linear regression analysis was conducted to assess both direct and moderating effects. In the first step the model considered the two predictors, sex (coded as female = 0, male = 1) and age (standardized as a z-score); then, in the second step, the interaction term between sex and age was added. The total score on the SMFQ served as the dependent variable. Furthermore, based on the regression results, for each score, descriptive statistics were computed for the overall sample and stratified by sex, and normative reference values were established to facilitate score interpretation, identifying as a function of the sex and age the expected value (mean) and threshold values corresponding to the 84th, and 95th percentiles.

## Results

### Confirmatory factor analysis

Results of the *CFA* confirmed that the 1-factor model had adequate fit indices, *RMSEA* =.074, 90%CI [.065;.083], *CFI* =.965, *TLI* =.957, *SRMR* =.058, *SB*χ^2^(64, *N* = 580) = 267.23, *p* <.001. Data showed that all items had a loading greater than.45. However, results from the two-factor model showed that this latter solution had better fit indices, *RMSEA* =.057, 90%CI [.047;.066], *CFI* =.980, *TLI* =. 975, *SRMR* =.048, *SB*χ^2^(63, *N* = 580) = 179.74, *p* <.001; and improved significantly the fit to the data, ΔSBχ^2^(1, *N* = 580) = 45.76; *p* <.001; ΔCFI = − .015. Considering the better indices of the two-factor model this latter model was considered as the optimal solution. In particular, all items showed loadings greater than.51 for the first factor related to ‘affective-somatic’ symptoms, and greater than 0.46 for the second factor related to “cognitive” symptoms. Moreover, a high correlation, *r* =.85, among the two factors was found, suggesting that they can be considered as the expression of a single higher-order factor.

#### Measurement invariance for sex

The first step was to test configural invariance. A simultaneous two-factor multi-group *CFA* model of mean and covariance structure was tested in males and females on the 13-item SMFQ. This model (Model A) does not impose any equality constraints on the parameter estimates across groups, except that in both groups, for each factor, the mean was fixed to 0 and the variance to 1, and the uniqueness were constrained to 1 for identifying the model. The results indicated a good fit of the tested model, *RMSEA* = 0.054, *CFI* = 0.976, *TLI* = 0.971, *SRMR* = 0.057, *SB*χ^2^(126, *N* = 580) = 232.93, *p* <.001 (see Table 1). The second step was to test metric invariance. In addition to the previous model, in this step the slopes of the items on the respective factors were constrained to be equal across groups. In this model (Model B) the factor means were fixed to 0 and the variance to 1 in both groups, and the uniqueness were constrained to 1 for model identification purposes. The results showed that the model fitted the data well, *RMSEA* = 0.043, *CFI* = 0.983, *TLI* = 0.981, and *SRMR* = 0.065, *SB*χ^2^(139, *N* = 580) = 214.09, *p* <.001 (see Table 1); and that the constraints did not significantly reduce the model fit, Δ*SB*χ^2^(13, *N* = 580) = 16.06, *p* =.246, ∆*CFI* = − 0.007. The subsequent step was to test the scalar invariance. In addition to the previous model, in this step all item thresholds were constrained to be equal across groups (Model C). In this model the factor means were fixed to 0 and the variance to 1, and the uniqueness were constrained to 1 for model identification purposes only in the first group, while they were freely estimated in the second group, allowing for more flexibility in the model. The results indicated that the model fit the data well, *RMSEA* = 0.064, *CFI* = 0.961, *TLI* = 0.959, and *SRMR* = 0.064, *SB*χ^2^(148, *N* = 580) = 325.60, *p* <.001. However, data indicated that the model exhibited a significant reduction in the fit compared to Model B, Δ*SB*χ^2^ (9, *N* = 580) = 64.15, *p* <.001, ∆*CFI* = 0.022. The modification indices analysis suggested freely estimating the thresholds of item 6. The new partial scalar invariance model (Model C2) showed a good fit to the data, *RMSEA* = 0.054, *CFI* = 0.972, *TLI* = 0.970, and *SRMR* = 0.061, *SB*χ^2^(146, *N* = 580) = 270.78, *p* <.001 (see Table 1). Furthermore, the model did not cause a significant loss of fit compared to Model B, Δ*SB*χ^2^ (7, *N* = 580) = 1.90, *p* =.965; ∆*CFI* = 0.011. The final phase consisted of performing the strict invariance test. In addition to the previous model, in this step all residual variances of the items were constrained to be equal across groups (Model D). Given that the residual variances were initially fixed to 1 for identification purposes, we further constrained the residual variances of the second group to be 1, thereby ensuring that the residuals were equal across groups. The results indicated that the model fit the data well, *RMSEA* = 0.051, *CFI* = 0.973, *TLI* = 0.973, and *SRMR* = 0.063, *SB*χ^2^ (159, *N* = 580) = 281.22, *p* <.001; and that the model did not cause a significant loss of fit compared to Model C2, Δ*SB*χ^2^ (13, *N* = 580) = 24.71, *p* =.025; ΔCFI = 0.

**Table 1:** Results of measurement invariance analysis for sex and age as a function of the considered factor and the scale version (13-item and 12-item scale)

Model^^^	SBχ²	df	RMSEA	CFI	TLI	SBχ²_diff_	df_diff_	∆CFI
13-item scale								
Sex								
Model A	232.93^**^	126	0.054	0.976	0.971	−	−	−
Model B	214.09^**^	139	0.043	0.983	0.981	16.06	13^a^	− 0.007
Model C	325.60^**^	148	0.064	0.961	0.959	64.15^**^	9^b^	0.022
Model C2	270.78^**^	146	0.054	0.972	0.970	1.90	7^b^	0.011
Model D	281.22^**^	159	0.051	0.973	0.973	24.71^*^	13^d^	− 0.001
*** Age***								
Model A	239.83^**^	126	0.056	0.981	0.976	−	−	−
Model B	240.35^**^	139	0.050	0.983	0.981	26.60^*^	13^a^	− 0.002
Model C	307.91^**^	152	0.059	0.974	0.973	41.08^**^	13^b^	0.009
Model D	316.55^**^	165	0.056	0.975	0.976	28.03^*^	13^c^	− 0.001
**12-item scale**								
*** Sex***								
Model A	206.34^**^	106	0.057	0.975	0.969	−	−	−
Model B	185.41^**^	118	0.044	0.983	0.981	13.06	12^a^	− 0.008
Model C	244.99^**^	126	0.057	0.970	0.969	15.55	8^b^	0.013
Model D	263.43^**^	138	0.056	0.969	0.970	28.11^*^	12^c^	0.001
*** Age***								
Model A	199.63^**^	106	0.055	0.982	0.978	−	−	−
Model B	211.95^**^	118	0.052	0.982	0.980	27.41^*^	12^a^	0
Model C	264.80^**^	130	0.060	0.974	0.974	33.01^**^	12^b^	0.008
Model D	280.76^**^	142	0.058	0.973	0.975	29.36^*^	12^c^	0.001

Note. ^ Model A: two-factor configural invariance (CI); Model B: two-factor metric invariance (MI); Model C: two-factor scalar invariance (SI); Model C2: two-factor partial SI; Model D: two-factor strict invariance (SI); Sex: Males *n* = 317, Females *n* = 263; Age: G1 [14–16 years] *n* = 293, G2 [17–20 years] *n* = 287; * *p* <.05; **p <.001; ^a^ The reference model is Model A; ^b^ The reference model is Model B; ^c^ The reference model is Model C; ^d^ The reference model is Model C2

Taking these results and previous observations in the literature into account [31, 43], to identify a version of the scale that was fully invariant across sex, the analyses of invariance of the measure were repeated by excluding item 6. The findings from this second analysis revealed that the 12-item scale is fully invariant across sex (see Table 1).

#### Measurement invariance for age

In the first step, we tested the configural invariance across the two groups (Model A). The results indicated a good fit of the tested model, *RMSEA* = 0.056, *CFI* = 0.981, *TLI* = 0.976, *SRMR* = 0.055, *SB*χ^2^(126, *N* = 580) = 239.83, *p* <.001 (see Table 1). In the second step, we tested metric invariance (Model B). The results showed that the model fitted the data well, *RMSEA* = 0.050, *CFI* = 0.983, *TLI* = 0.981, and *SRMR* = 0.067, *SB*χ^2^(139, *N* = 580) = 240.35, *p* <.001 (see Table 1); and that the constraints did not significantly reduce the model fit, Δ*SB*χ^2^ (13, *N* = 580) = 26.60, *p* <.05, ΔCFI = 0.002. The next phase involved the scalar invariance test (Model C). Results showed a good fit to the data, *RMSEA* = 0.059, *CFI* = 0.974, *TLI* = 0.974, and *SRMR* = 0.060, *SB*χ^2^(153, *N* = 580) = 306.45, *p* <.001 (see Table 1); and that the constraints did not significantly reduce the model fit, Δ*SB*χ^2^(13, *N* = 580) = 41.08, *p* <.001, Δ*CFI* = 0.009. The last step was the strict invariance test (Model D). The results indicated that the model fit the data well, *RMSEA* = 0.056, *CFI* = 0.975, *TLI* = 0.976, and *SRMR* = 0.069, *SB*χ^2^ (165, *N* = 580) = 316.55, *p* <.001; and that the constraints did not significantly reduce the model fit, Δ*SB*χ^2^ (13, *N* = 580) = 28.03, *p* <.01, ΔCFI = − 0.001.

Moreover, in line with the analysis carried out for the sex invariance, the analyses of invariance of the measure were repeated by excluding item 6. The findings from this second analysis confirmed that the 12-item scale is fully invariant across ages (see Table 1).

#### Reliability

The reliability coefficients for the two dimensions of the SMFQ and the overall reliability coefficients of the scale were calculated. The values for the “affective-somatic” dimension (F1) were α = 0.78 and ωt = 0.86, while for the “cognitive” dimension (F2) were α = 0.84 and ωt = 0.88. The overall reliability coefficients of the scale were α = 0.87 and ωt = 0.89. These values indicate that the scale demonstrates good internal consistency.

#### Construct validity

Results of the correlation analysis confirmed that SMFQ is a valid instrument, showing a good concurrent, *rs* > 0.60, and divergent, *r* =.234, validity (see Table 2).

**Table 2:** Correlation between SMFQ, PAQ, SDQ and WSNN for nomological validity

Variables^	SMFQ	*N*	M (SD)
SDQ-I	0.666^***^	313	5.04 (3.56)
PAQ	0.606^***^	267	77.11 (14.60)
WNSS	0.234^***^	267	3.92 (0.72)

Note. *^* SMFQ = Short Mood and Feelings Questionnaire; SDQ-I = Internalising subscale from the Strength and Difficulties Questionnaire; PAQ = Personality Assessment Questionnaire; WNSS = Weinstein’s Noise Sensitivity Scale; ^***^p <.001

#### Effect of sex and age on SMFQ scores

Results showed that the model including sex and age as predictive factors influenced the perceived depression, *F*(2,577) = 58.504, *p* <.001, *R*^*2*^ = 0.169. Results showed a significant effect of both predictive factors. The sex effect indicated that, over and above the age, males reported a lower depression than females, *b* = -4.4, *beta* = − 0.374, *p* <.001. The age effect indicated that, over and above sex differences, the greater the age the greater the perceived depression, *b* = 0.702, *beta* = 0.110, *p* =.005. The inclusion of the sex by age interaction effect did not improve the fit of the model, *F*(1,576) = 0. 06, *p* =.806, *R*^*2*^_*diff*_ = 0. Completely equivalent results were obtained using the 12-item version of the scale. In Table 3, threshold values, corresponding to the 84th, and 95th percentiles as a function of the sex and age, were reported (see Table 3).

**Table 3:** Normative reference values of SMFQ total score as a function of sex and age

Age	Expected value	84th percentiles	95th percentiles
Males			
14 years	5.0	10.0	13.0
15 years	5.0	10.0	14.0
16 years	5.0	11.0	14.0
17 years	6.0	11.0	15.0
18 years	6.0	12.0	15.0
19 years	7.0	12.0	15.0
20 years	7.0	12.0	16.0
**Females**			
14 years	9.0	14.0	18.0
15 years	9.0	15.0	18.0
16 years	10.0	15.0	19.0
17 years	10.0	16.0	19.0
18 years	11.0	16.0	19.0
19 years	11.0	16.0	20.0
20 years	12.0	17.0	20.0

Note. *^* SMFQ = Short Mood and Feelings Questionnaire

To further support the normative data, Table 4 presents descriptive statistics of SMFQ total scores for the overall sample as well as separately for males and females.

**Table 4:** Descriptive statistics of SMFQ total score in the total sample and as a function of the sex

Sample	*N*	Mean	SD	Min	Max	Skew	Kurtosis
Males	317	5.99	5.05	0	25	1.23	1.31
Females	263	10.65	5.75	0	25	0.45	-0.67
Total	580	8.11	5.85	0	25	0.77	-0.16

## Discussion

This study aimed to investigate the psychometric properties of the Italian version of the SMFQ. The primary objective was to verify the factor structure. Based on existing literature, both one- and two-factor models were tested. Confirmatory factor analysis indicated that the 1-factor model had a good fit, consistent with previous studies [24, 29, 31, 33]. However, the two-factor structure exhibited better fit indices. This finding aligns with previous research by Lewis et al. [36], which suggests that a two-factor model distinguishing between affective-somatic and cognitive symptoms, despite their high correlation, may more accurately describe depressive symptomatology. Neurobiological evidence further supports the validity of this two-factor structure, indicating distinct correlates for the affective and cognitive domains [37–39]. However, it is important to note that the high correlation between the two factors [36] suggests that, although they are distinct, they reflect a single overarching construct of depression. Recognizing these distinct symptom domains is crucial for a comprehensive understanding of the disorder. Although this distinction may seem primarily relevant for statistical purposes rather than direct clinical application, it has important clinical implications. In particular, it highlights the need to assess affective-somatic symptoms as potential early indicators of depression alongside cognitive symptoms. Research suggests that patients diagnosed with depression often first report somatic and affective symptoms before cognitive ones [65]. Additionally, adolescents with depression tend to exhibit more somatic symptoms than their healthy peers. However, despite their strong association with depression, these symptoms often go unnoticed [66–67]. Studies have also shown that primary care physicians do not always recognize depression in patients presenting with multiple unexplained somatic symptoms. Instead, they are more likely to suspect depression when patients repeatedly seek medical consultations for persistent symptoms [68]. Since depressed adolescents with somatic symptoms are more likely to seek medical help for physical complaints rather than psychological distress [69], there is a risk that depression may remain masked, delaying appropriate treatment [66]. Therefore, while the distinction between affective-somatic and cognitive symptoms does not necessarily imply different treatment approaches, careful evaluation of somatic symptoms in paediatric settings remains crucial. Since these symptoms often serve as early warning signs of depression, recognizing them can facilitate earlier diagnosis and intervention.

The second objective was to assess the measurement invariance of the scale across sex and age. Our results showed a full invariance of the 13-item version of the scale with respect to age, in line with the existing literature [43]. This shows that the Italian version of the SMFQ can be used in subjects between 14 and 20 years old by measuring depressive symptoms in an age invariant manner. It must be emphasised that our data are cross-sectional and that it would therefore be interesting to confirm the invariance with respect to age of the Italian version of the SMFQ through longitudinal studies. As concern sex, our results showed a partial invariance of the 13-item version of the scale. In particular, in line with previously reported literature [43], males and females showed a different item intercept for the item 6 (“I cried a lot”). Based on previous studies [44, 45, 47, 48], it is possible to state that this result reflects the knowledge that females generally exhibit higher levels of depression and are more prone to crying or admitting to crying than males when they feel sad [51]. Therefore, in accordance with another previous study [31], our results confirm that one item perform differently for the two sexes, at the same level of depression, that is females had a lower threshold for crying than males. Considering this evidence and recognizing that item 6 was identified as a biased source of variability in responses between males and females, a subsequent measurement invariance analysis was conducted excluding item 6 on the 12-item version of the scale. The results of this second analysis confirmed a full MI of the 12-item scale across both sex and age. However, it should be noted that the analysis excluding item 6 was undertaken on an exploratory basis, prompted by the previous literature [31–43] and the outcomes of the measurement invariance analyses, with the aim of gaining a deeper understanding of its contribution to sex-based differences. The validated Italian version of the SMFQ includes all 13 items, and item 6 remains a relevant component of the scale, given that crying is a clinically significant symptom of depression in adolescents. Rather than recommending its exclusion, the present findings highlight the importance of exercising caution when comparing total scores across sexes, as this item may contribute to a modest but significant degree of measurement bias. Future research may consider whether alternative formulations of this item could mitigate differential functioning while preserving its clinical relevance.

The third objective was to measure the reliability and validity of the Italian version of the SMFQ. The scale revealed a high level of internal consistency and coherence, as evidenced by the Cronbach’s alpha and the Omega values. That is, the nomological validity of the scale was confirmed. In particular, a good convergent validity was observed, as shown by the strong correlation with the psychological maladjustment measure PAQ and with the “internalising” scale of SDQ. Divergent validity was also supported by the weak correlation observed between the SMFQ and the WNSS. The WNSS was chosen as it measures environmental reactivity which is conceptually distinct from the cognitive and affective symptoms of depression. Although the existing literature acknowledges some overlap between depression and noise sensitivity, we selected the latter due to its conceptual distinctiveness [see 70–71]. Specifically, while both constructs may share underlying neurobiological vulnerabilities (e.g., amygdala structure and function, hippocampus, brain volume), depression is a multidimensional clinical syndrome, whereas noise sensitivity is best understood as a sensory-emotional trait or temperament reflecting heightened reactivity to environmental stimuli (e.g., Environmental Sensitivity; see [72]). This distinction is empirically supported by the observed correlation between the two constructs, which, although statistically significant, is modest in magnitude. The shared variance between the two measures is approximately 5.5%, indicating a sufficient degree of independence for the purposes of discriminant validity assessment, in line with established psychometric standards.

Lastly, another aim of this study was to establish reference values, including expected values (mean) and threshold values corresponding to the 84th and 95th percentiles, to support the interpretation of test scores, providing a framework for incorporating the SMFQ into clinical practice. Accordingly, scores falling between the mean and the 84th percentile can be considered within the typical range; those above 84th requires clinical attention and possibly a referral to child and adolescent mental health services (camhs) and those above 95th percentiles require close monitoring and a quick referral camhs. It is worth to notice, that although these reference values do not have any diagnostic significance, they can be particularly useful in clinical practice for identifying cases that may require further evaluation or for gaining insight into adolescents’ self-perception of depressive symptoms. Thus, while the SMFQ is a valid screening tool for self-reported depressive symptoms, it should not be considered a substitute for a comprehensive clinical assessment or for diagnostic purposes.

## Limitation

Although this validation study significantly contributes to the current scientific literature on depressive screening in Italian adolescents, it has some limitations. Notably, we did not collect data from a clinical sample nor use clinical interviews or preexisting diagnoses to establish criterion validity. Consequently, the Italian version of the SMFQ should not be considered a diagnostic tool. While the SMFQ has shown good discriminative power between clinical and non-clinical samples in other languages (e.g. [25, 23, 73]), this study does not provide evidence of its sensitivity and specificity, nor does it offer a diagnostic cut-off score for the Italian version. Future studies should aim to address these limitations by including clinical samples and employing clinical interviews or established diagnoses to better establish the criterion validity of the Italian SMFQ. Additionally, further research is needed to determine the sensitivity, specificity, and appropriate diagnostic cut-off scores for the Italian adolescent population. This would enhance the utility of the SMFQ as a screening tool and potentially as part of a comprehensive diagnostic process. In this regard, it is also important to highlight that the regression analyses were conducted not for predictive purposes, but rather to assess whether normative scores required adjustment based on two variables previously considered for the measurement invariance testing. Consequently, the model may not fully adhere to the assumptions required for correct parameter estimation, such as comprehensive model specification and the inclusion of all relevant covariates, which may further limit the interpretability and generalisability of the findings. Future research should consider employing more robust modelling approaches to explore these associations further and to validate the need for normative score adjustments.

An additional limitation of this study is based on the limited number of schools from which the participants were recruited; this may not be fully representative of the Italian population, in terms of ethnicities and socio-economic status. Future studies should seek to validate the SMFQ in more varied samples, including adolescents from various Italian regions and socioeconomic backgrounds, to confirm its applicability on a national scale. Additionally, as data were collected during the early phase of the COVID-19 pandemic in Italy, the emotional impact of that context may have influenced participants’ responses. This should be considered when interpreting the findings and assessing generalizability.

Finally, we did not carry out the test-retest reliability assessment, which is crucial for establishing the consistency of the SMFQ over time. Although a previous study has examined the test-retest reliability of the SMFQ in other languages, reporting satisfactory results [74], this analysis was not conducted in our study. Therefore, future research should address this gap by assessing the test-retest reliability of the SMFQ in the Italian adolescent population, further strengthening its validity as a screening and monitoring tool.

## Conclusions

In general, our results suggest that the Italian version of the SMFQ is a reliable and valid instrument to readily identify depression among Italian-speaking adolescents aged between 14 and 20 years. Due to its simplicity, speed of administration and solid psychometric properties, this instrument can be easily applied in epidemiological studies, such as depression prevention screenings among adolescents. Furthermore, the SMFQ could be effectively integrated into routine paediatric health assessments. However, it is essential to recognize that the scores provided by the SMFQ are only suggestive rather than diagnostic; they do not represent clinical cut-off points but serve as useful criteria for guiding further evaluation. If an adolescent scores above the average range on the SMFQ, paediatricians and other operators should refer the adolescent to local camhs for a comprehensive diagnostic assessment. Indeed, using the SMFQ in non-clinical settings, such as schools, or within routine medical assessments, can help remove financial or stigma-related barriers that often hinder access to mental health services. This can play a significant role in improving the identification and treatment of depressive symptoms in adolescents. Further studies will be needed to assess the potential use of this instrument for diagnostic purposes, including the identification of clinically relevant cut-off scores.

## Supplementary Information

Below is the link to the electronic supplementary material.

Supplementary Material 1

## Data Availability

The datasets used and/or analysed during the current study are available from the corresponding author on reasonable request.
