# Supplementary material for: Validation of the Italian version of the Short Mood and Feelings Questionnaire (SMFQ) in a sample of adolescents
Source: Ital J Pediatr. 2026 Mar 21;52:73. doi: 10.1186/s13052-026-02240-7 (PMC13130510; doi:10.1186/s13052-026-02240-7)
Supplement: Supplementary file 1 — Supplementary Material 1 [file 13052_2026_2240_MOESM1_ESM.docx]

**Italian Version of the SMFQ**

**Instructions:** Adesso ti presentiamo alcune affermazioni che descrivono modo di sentirsi o di agire dei/lle ragazzi/e della tua età. Per ciascuna frase, ti chiediamo di rispondere indicando quanto bene ciascuna frase descrive come tu ti sei sentito/a o hai agito in queste **ultime due settimane**. Per favore rispondi utilizzando una di queste risposte: **“non vero”**, **“qualche volta vero”** oppure **“vero”**. Rispondi nel modo più sincero possibile sapendo che non ci sono risposte giuste o sbagliate ma solo risposte che sono vere per te.

|  | Non vero | Qualche volta vero | Vero |
| --- | --- | --- | --- |
| 1. Mi sono sentito/a infelice o triste |  |  |  |
| 2. Nulla mi è piaciuto veramente |  |  |  |
| 3. Mi sono sentito/a così stanco/a che sono stato/a seduto/a senza fare niente |  |  |  |
| 4. Mi sono sentito/a molto irrequieto/a |  |  |  |
| 5. Mi sono sentito/a non più capace |  |  |  |
| 6. Ho pianto molto |  |  |  |
| 7. Ho trovato difficile concentrarmi e pensare adeguatamente |  |  |  |
| 8. Ho odiato me stesso/a |  |  |  |
| 9. Sono stato/a una cattiva persona |  |  |  |
| 10. Mi sono sentito/a solo/a |  |  |  |
| 11. Ho pensato che nessuno mi voglia veramente bene |  |  |  |
| 12. Ho pensato di non poter essere bravo/a e capace come gli altri ragazzi/e |  |  |  |
| 13. Ho fatto tutto male |  |  |  |
